# Generation of a Novel Mesothelin-Targeted Oncolytic *Herpes* Virus and Implemented Strategies for Manufacturing

**DOI:** 10.3390/ijms22020477

**Published:** 2021-01-06

**Authors:** Guendalina Froechlich, Chiara Gentile, Luigia Infante, Carmen Caiazza, Pasqualina Pagano, Sarah Scatigna, Gabriella Cotugno, Anna Morena D’Alise, Armin Lahm, Elisa Scarselli, Alfredo Nicosia, Massimo Mallardo, Emanuele Sasso, Nicola Zambrano

**Affiliations:** 1 CEINGE Biotecnologie Avanzate S.C.aR.L., Via G. Salvatore 486, 80145 Naples, Italy; froechlich@ceinge.unina.it (G.F.); chiara.gentile@unina.it (C.G.); pasqualina.pagano2@unina.it (P.P.); sarahscatigna@gmail.com (S.S.); nicosia@ceinge.unina.it (A.N.); 2 Dipartimento di Medicina Molecolare e Biotecnologie Mediche, Università degli Studi di Napoli Federico II, Via Pansini 5, 80131 Naples, Italy; carmen.caiazza@unina.it (C.C.); massimo.mallardo@unina.it (M.M.); 3 Nouscom S.R.L., Via di Castel Romano 100, 00128 Rome, Italy; luigia.infante@ibbc.cnr.it (L.I.); g.cotugno@nouscom.com (G.C.); m.dalise@nouscom.com (A.M.D.); a.lahm@nouscom.com (A.L.); e.scarselli@nouscom.com (E.S.)

**Keywords:** oncolytic virus, triple negative breast cancer, malignant mesothelioma, targeted therapy, MSLN

## Abstract

Background: HER2-based retargeted viruses are in advanced phases of preclinical development of breast cancer models. Mesothelin (MSLN) is a cell-surface tumor antigen expressed in different subtypes of breast and non-breast cancer. Its recent identification as a marker of some triple-negative breast tumors renders it an attractive target, presently investigated in clinical trials employing antibody drug conjugates and CAR-T cells. The availability of MSLN-retargeted oncolytic viruses may complement the current immunotherapeutic panel of biological drugs against HER2-negative breast and non-breast tumors. Methods: A fully virulent, tumor-targeted oncolytic *Herpes simplex* virus-1 (MSLN-THV) with a selectivity for mesothelin-expressing cancer cells was generated. Recombineering technology was used to replace an essential moiety of the viral glycoprotein D with antibody fragments derived from clinically validated MSLN monoclonal antibodies, and to allow IL12 cargo expression in infected cells. Panels of breast and female reproductive system cell lines were used to verify the oncolytic potential of the viral constructs. A platform for production of the retargeted viruses was developed in HEK 293 cells, providing stable expression of a suitable chimeric receptor. Results: We demonstrated the selectivity of viral infection and cytotoxicity by MSLN-retargeted viruses in a panel of mesothelin-positive cancer cells, originating from breast and female reproductive system tumors. We also developed a second-generation oncolytic MSLN-THV, encoding IL12, to enhance the immunotherapeutic potential of the viral backbone. A non-tumor cell line expressing a chimeric MSLN/Nectin-1 receptor, de-sensitized from antiviral responses by genetic inactivation of the Stimulator of Interferon Genes (*STING*)-dependent pathway was engineered, to optimize viral yields. Conclusions: Our proof-of-concept study proposes MSLN-retargeted herpesviruses as potential cancer immunotherapeutics for assessments in preclinical models of MSLN-positive tumors, complementing the available panel of oncolytic viruses to HER2-negative breast tumors.

## 1. Introduction

Over the last two decades, the deep molecular characterization of tumors led to the development of targeted therapeutics for individualized approaches in cancer therapy. The foundation of cancer precision medicine is the selection of an optimal targetable tumor associated antigen (TAA) that should be selectively expressed by cancerous cells and absent in healthy tissues and organs. Monoclonal antibodies and antibody fragment-based therapeutics (e.g., BiTEs, CAR-T cells, Antibody Drug Conjugates) represent the most implemented tools in targeted therapy. A potential drawback of targeted therapy is the acquired resistance that relies on cancer evolution and immunoediting that drive the loss of target expression under the selective pressure of the treatment. To overcome this limitation, a stronger contribution of the immune system is desirable, to elicit antitumor immunity against the targeted TAA and an epitope spreading to different tumor antigens and neoantigens. Oncolytic viruses (OVs) are emerging immunotherapeutics able to kill selectively cancerous cells, leaving healthy tissues undamaged [1,2,3,4,5]. The foundation of oncovirotherapy is currently established to rely on pleiotropic mechanisms, based on both cancer cell lysis and elicitation of systemic antitumor immunity leading to abscopal effect. The immunogenic way by which cancer cells succumb to viruses, namely immunogenic cell death (ICD), is actually the core activity of OVs [1,6]. Indeed, the OV-mediated lysis of cancer cells induces the release of tumor antigens that are captured by antigen presenting cells (APCs) that also “sense” the damage- and pathogen-associated molecular patterns (DAMPs, PAMPs), becoming activated. Engulfed antigens are processed and presented to adaptive immune compartment awakening the effector functions of anergic T lymphocytes and activating new anti-tumor reactiveness [7]. With the approval of Imlygic™ (T-VEC, Talimogene laherparepvec), many clinical trials have arisen, to treat different cancer indications as single agents, as well as in combination with checkpoint inhibitors [8,9,10,11,12,13]. Except for a few examples of viruses with a natural tropism for receptors overexpressed in tumors, OVs may typically infect both cancerous and healthy cells [14]. Even though the OV’s abortive infection of healthy tissues may be well tolerated (thanks to attenuating modifications within viral genomes), it limits the opportunity to use high doses for systemic delivery [15]. Targeted oncolytic viruses offer the possibility to implement fully virulent viruses with a tropism redirected to any tumor antigen of interest. *Herpes simplex* type 1 (HSV-1) is one of the most implemented OVs for retargeting, thanks to the extensive knowledge of viral receptors and the easiness to manipulate its large genome [7,16,17,18,19,20,21,22]. Similarly to CAR-T cells, targeted *Herpes* viruses (THVs) can be potentially redirected to any tumor antigen of interest, by substitution of an essential moiety of viral glycoproteins (i.e., gD) with a TAA-targeting antibody fragment (scFv). The use of THVs allows to exploit the best of both targeted therapy and cancer immunotherapy, as recently demonstrated in preclinical combination settings with PD-1 inhibitor [7,23]. The self-origin of such tumor associated antigens (e.g., HER2) increases the potential risk of deleterious on-target, off-tumor toxicity; for these reasons, we recently published a proof-of-concept of dual restriction by combining the retargeting to HER2 to replicative conditioning to cancer cells by the SurE_oHSV hosting surviving promoter driving ICP4 expression [7].

Human mesothelin (MSLN) is a tumor associated antigen overexpressed in several aggressive, poor prognosis, and orphan-drug tumors. MSLN was discovered in the 1990s by Ira Pastan and Mark Willingham as a marker for human malignant mesotheliomas and ovarian cancers recognized by the K1 antibody. Immunohistochemical studies confirmed the limited expression of MSLN in healthy tissues (mesothelial cells of the pleura, pericardium, peritoneum, fallopian tubes, and tonsils), underlining the potential to exploit it as target for cancer therapy [24]. Some years later, MSLN was revealed as broadly overexpressed in many others cancer indications, including squamous cell carcinomas of the esophagus, pancreas, lung, stomach, bile ducts, colorectal, and breast cancer, where its expression correlates with a worse prognosis [25,26,27,28]. MSLN is a glycophosphatidylinositol (GPI)-anchored plasma membrane glycoprotein synthesized as a 71 kDa precursor, rapidly cleaved in its N terminus domain. The resulting 40 kDa C-terminal protein anchored to the cell membrane is known as mature MSLN, whereas the shed N-terminal fragment is the megakaryocyte potentiating factor (MPF) [29]. Recently, a soluble form of mature MSLN (soluble mesothelin-related protein, SMRP) derived by alternative splicing or protease cleavage, was also reported, and it is currently assessed as a tumor biomarker [30,31,32].

Currently, MSLN-targeted therapies are investigated in many clinical trials for different solid tumor indications using different agents including CAR-T cells, monoclonal antibodies (e.g., Amatuximab, MORAb-009), drug conjugates, Immunotoxins (e.g., SS1P), T cell-engaging bispecific antibody (BiTEs) and cancer vaccines [33,34,35,36,37,38,39,40]. To date, clinical trials testing biodistribution, toxicity, and objective response of MSLN-targeted therapeutics showed no severe adverse effects and promising efficacy spurring clinicians to further explore this target in phase II clinical trials and combination studies [25,33,41,42].

Among the cancer indications expressing MSLN, we focused our efforts on breast and female reproductive system tumors. The poor prognosis and the lack of targets (e.g., hormonal and HER2) amenable by therapeutic antibodies or hormone blocking drugs, still makes triple-negative breast cancer (TNBC) a major clinical challenge. MSLN has a high level of expression in a large percentage (up to 63%) of TNBC, in which it promotes invasion and metastasis, and correlates to decreased disease-free survival [43,44,45]. Accordingly, mesothelin may represent a novel target, complementary to HER2-based THVs, to be added to the growing arsenal of retargeted oncolytic viruses to treat breast as well as, in principle, any mesothelin-positive cancer.

The current success of OVs, especially as sensitizer to immune checkpoint inhibitors (e.g., PD-1, PD-L1, CTLA-4), could in a next future pave the way to a broadened clinical use, raising the issues of: (i) reducing costs and (ii) improving viral yield for high-titer testing [1,7,11,12,13,46]. Thus, the implementation of an efficient virus manufacturing strategy is desirable. We recently demonstrated that the genetic material of viruses is probably the main pathogen associated molecular pattern (PAMP) recognized by intracellular Pattern Recognition Receptors (PRR) [47]. Interestingly, beyond toll-like receptors (TLRs) expressed by specialized immune cells, emerging evidence is revealing novel constitutive PRR genes widely expressed in all tissues and organs. We and others revealed that the sensing of viral DNA by STING axis (cGAS, STING, TBK1, IRF3) restricts the replication and maturation of HSV-1 [48,49,50,51,52].

In the present paper, we describe the generation of MSLN-targeted herpes oncolytic viruses engineered through the insertion of different antibody fragments within the viral glycoprotein D; the selected backbone was also improved by adding a IL12 cargo as an adjuvant transgene to increase the immunolytic potential of MSLN^+^ tumors. By functionally inactivating *STING* gene in HEK 293 cell line we generated a virus high-yielding cell line for oncolytic virus manufacturing. By further engineering the *STING* KO cell line with a chimeric non-secretable MSLN receptor for proper MSLN-THV manufacturing, our proof-of-concept study proposes MSLN-THV as potential candidates for preclinical evaluation of breast cancer models [53,54,55].

## 2. Results

### 2.1. Generation of Oncolytic Herpes Viruses with a Specific Tropism for Human Mesothelin

**Figure 1:**
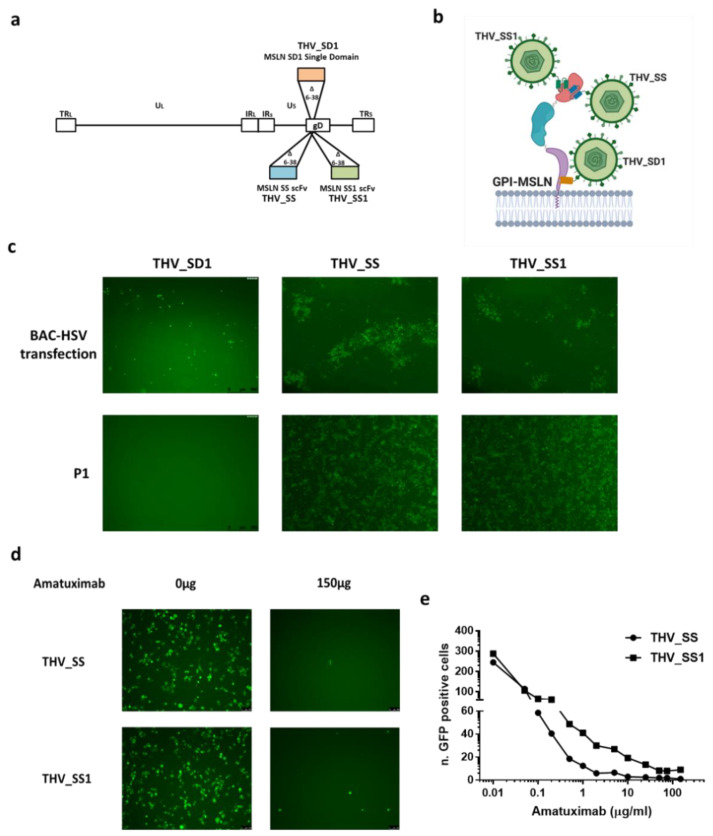
Generation of *Herpes simplex* type 1 (HSV-1)-based oncolytic viruses retargeted to Mesothelin (MSLN). (**a**) Three targeted *Herpes* viruses (THVs) were generated by substitution of the essential moiety of viral glycoprotein D (aa 6 to 38) with three different antibody fragments recognizing MSLN: SD1 (orange), SS (light blue), and its affinity matured form SS1 (green) (**b**) targeting two different domains of MSLN. (**c**) THV-SS, THV-SS1, and THV-SD1 Bacterial Artificial Chromosome (BAC) DNAs were transfected in SKOV3 cells to produce infectious viral particles (top panels); THV-SD1 failed to be recovered in first passage of infection (P1) (bottom panel). (**d**) Viral infection and spread were analyzed in presence of the monoclonal antibody Amatuximab (α-MSLN) used at saturating concentration (150 μg) to compete with THV-SS and THV-SS1 viruses. (**e**) The Amatuximab antibody was used at increasing concentrations and the number of eGFP-positive (infected) cells was determined. The statistical significance was calculated by Student’s *t*-test and resulted *p* < 0.05 in the range of Amatuximab concentration between 0.1 and 50 μg/mL.

In the effort to generate an oncolytic *Herpes simplex* Virus 1 retargeted to mesothelin-expressing cells, we searched scientific literature for characterized antibody fragments (scFv_s_), useful to retarget HSV-1 virus to human MSLN, by selecting the most appropriate protein epitope for viral entry; the insertion site for the scFv_s_ was selected into a well characterized viral glycoprotein D (gD) deletion (aa 6 to 38), which was proven to be effective for HER2 retargeting [7,17,18]. Mature MSLN is structurally divided into three regions: starting from N- to C-terminuses (N-terminus region I, intermediate region II, juxtamembrane region III). Despite most of the MSLN-specific antibodies recognizing region I, the membrane proximal region III may also represent an attractive target, as revealed in studies developing CAR-T and cytotoxic antibodies formats [56,57]. To target region I, we selected SS scFv and its affinity-matured derivative SS1, based on the validated efficacy and safety of their immunotoxin (SS1-immunotoxin, SS1P) and antibody (MORAb-009, Amatuximab) formats in human cancer patients bearing MSLN^+^ tumors [29,42,58,59,60,61]. In order to target region III of MSLN, we hypothesized that the docking of a retargeted virus to such cell surface-proximal epitope could have facilitated the interactions between viral glycoproteins (gH/gL and gB) and host cell ligands for the fusion of the viral envelope to host cell membrane [62]. We selected the human single-domain (VH) SD1, instead of a full scFv, to facilitate the access to the epitope, in a position very close to the cell surface [63]. Starting from a wild-type recombinant BAC-*Herpes simplex* Virus 1 (R-LM55), the three THVs were obtained by recombineering through the substitution of the essential moiety of viral glycoprotein D (aa 6 to 38) with the three different antibody fragments SS, SS1, and SD1 (Figure 1a,b) [7,17,18]. This modification in gD was demonstrated to confer both the complete detargeting from endogenous ligands of HSV-1, and proper retargeting to the selected TAAs. THV_SD1, THV_SS, and THV_SS1 BAC DNAs were transfected in SKOV3 cell line to recover infectious viral particles (passage 0, P0). The reporter eGFP gene, inserted within BAC region under the control of an immediate early viral promoter, was useful to monitor the viral infection and spread. The fluorescence images in Figure 1c show the presence of sparse single cells (SD1) or clustered plaques of eGFP-positive populations (SS and SS1), suggesting a better performance of THV_SS and THV_SS1, compared to THV_SD1. The infection of freshly seeded SKOV3 cells (P1) with cell lysates obtained from P0 samples confirmed the inability of SD1 antibody fragment to mediate an effective viral entry, presumably due to steric hindrance of viral particle (Figure 1c). THV_SS and THV_SS1 were successfully recovered, as inferred by eGFP expression by the whole cell monolayer at P1 (Figure 1c).

To confirm that infection of SS_ and SS1_THVs was actually occurring via mesothelin, a neutralization assay was carried out with saturating amount of the α-MSLN monoclonal antibody Amatuximab for competition with THV_SS and THV_SS1 challenge (Figure 1d) [60,61]. The inhibition of infection by Amatuximab confirmed the THV_SS- and THV_SS1-retargeting to human mesothelin (Figure 1d).

Next, we asked whether the improved affinity of SS1 to MSLN, compared to SS scFv [61], could have enhanced the ability of the corresponding virus to infect cancer cells. Thus, cells were challenged to increasing concentrations of Amatuximab and, after 2 h of incubation, they were infected with THV_SS1 at 1 MOI. The number of eGFP-positive (infected) cells was analyzed 24 h post infection (Figure 1e). Although the neutralization by Amatuximab at the >0.1 µg/mL concentration range was highly effective for both viruses, THV_SS1 showed to be able to infect more cells, compared to THV_SS, thus confirming an increased affinity to MSLN, also in the context of a targeted *Herpes* viral backbone.

**Figure 2:**
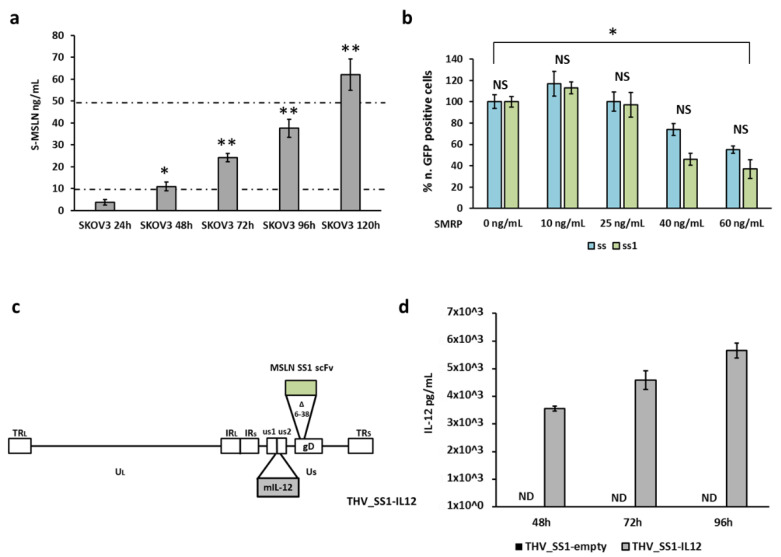
Soluble MSLN does not interfere with virus spread and mIL12 production. (**a**) Secreted soluble MSLN in supernatant of SKOV3 cell line was quantified by ELISA assay up to 120 h post seed. Dashed lines define the range of soluble mesothelin (SMRP) concentration in tumor patients. The statistical significance was calculated by Student’s *t*-test and resulted *p* < 0.05 * comparing 24 and 48 h, *p* < 0.005 ** comparing 48 and 72 h/72 and 96 h/96 and 120 h. (**b**) The same conditioned media described in panel A were used to infect fresh SKOV3 cells with THV_SS (light blue) and THV_SS1 (green) resulting in limited alteration of viral entry at the highest SMRP concentrations. The statistical significance was calculated by Student’s *t*-test and resulted not significant (NS) comparing THV_SS and THV_SS1 at each time point and *p* < 0.05 * comparing both THV_SS and THV_SS1 at 0 and 60 ng/mL. (**c**) Schematic cartoon of second generation THV_SS1 encoding mIL-12 (THV_SS1-IL12); the mIL-12 expression cassette was inserted into the intergenic region US1/US2. (**d**) Production of mIL-12 cytokine quantified by ELISA assay from 48 to 96 h.

Mature MSLN is actively secreted in fluids (ascites and blood) of tumor-bearing patients, as the result of aberrant splicing or juxtamembrane cleavage (soluble mesothelin related protein, SMRP) [30]. The possible competition of SMRP with the cell-anchored MSLN could, accordingly, contribute to neutralization of MSLN-targeting therapeutics [38,39]. For this reason, we verified the presence of soluble MSLN in cancer cell supernatants and its impact on viral infection and spread as, in principle, SMRP could occupy and neutralize scFv_s_ on THV’s envelope. SMRP was quantified from SKOV3 cell supernatants, demonstrating a remarkable accumulation over time, from 1 to 5 days after cell seeding (Figure 2a). Interestingly, SMRP concentration into the cell supernatant was comparable to those observed in body fluids as diagnostic and prognostic tumor biomarker (10 to 50 ng/mL) [64,65]. To assess the impact of soluble MSLN on THVs’ infection, the conditioned supernatants from SKOV3 cells containing increasing concentration of SMRP (Figure 2a) were used to precondition viral particles before infection of freshly seeded SKOV3 cells. To this end, THV_SS and THV_SS1 were pre-incubated for 2 h with SMRP-containing media to allow SMRP to interact with scFv on THV envelope. The impact of SMRP on mesothelin THVs’ entry was assessed as number of eGFP positive cells 24 h post-infection and expressed in terms of percentage compared to viruses pre-incubated with non-conditioned medium (0 ng/mL SMRP). As for CAR-T cells, only the highest concentrations of SMRP slightly affected THV infection; thus, both THV_SS and THV_SS1 retained their ability to efficiently infect MSLN^+^ cells in the presence of the soluble mesothelin (SMRP) at the concentrations observed in cancer patients. Interestingly, no statistically relevant differences were observed between THV_SS and THV_SS1 (Figure 2b). The same SMRP-competition experiment was performed with parental non-retargeted R-LM55 virus that resulted, as expected, not affected by SMRP (data not shown).

An emerging approach to maximize the efficacy of oncolytic viruses involves arming them with payloads to enhance antitumor immunity [1]. We and others recently demonstrated that interleukin 12 (IL12) significantly enhanced the antitumor efficacy of a retargeted oncolytic virus through T cell activation [23,66]. Based on these evidences, we devised an arming strategy to insert IL12 into the intergenic site between viral US1 and US2 genes of THV_SS1 (Figure 2c) as preparatory for preclinical translation. Quantification of the secreted cytokine by the newly generated THV_SS1-IL12 virus resulted in the actual accumulation of the functional mIL12 p40/p35 heterodimer, as revealed by ELISA assays (Figure 2d).

### 2.2. THV_SS1 Exerts Mesothelin-Dependent Cytotoxicity in Human Cells

To correlate the specificity of viral entry into host cells to cellular toxicity, we searched for a cell line not expressing human MSLN, in which to obtain stable expression of the tumor antigen. This approach was aiming to generate a pair of genetic background-matched cell lines for clear attribution of the cytotoxicity to MSLN. HEK293 cells, physiologically negative for MSLN [67], were proven not to express endogenous MSLN (data not shown); thus, they were stably transduced with exogenous MSLN to allow infection by THV_SS1.

**Figure 3:**
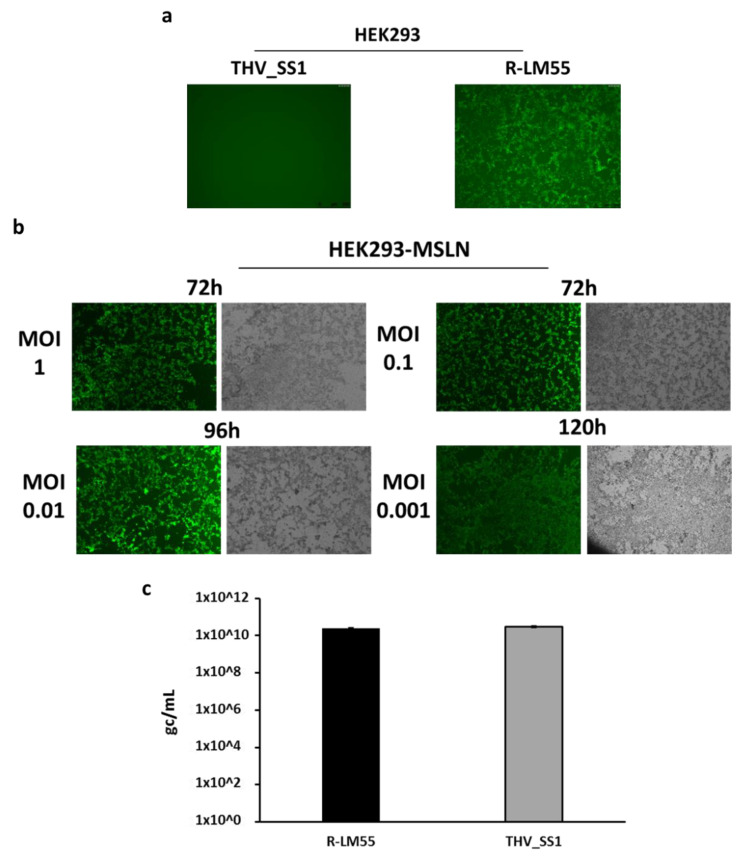

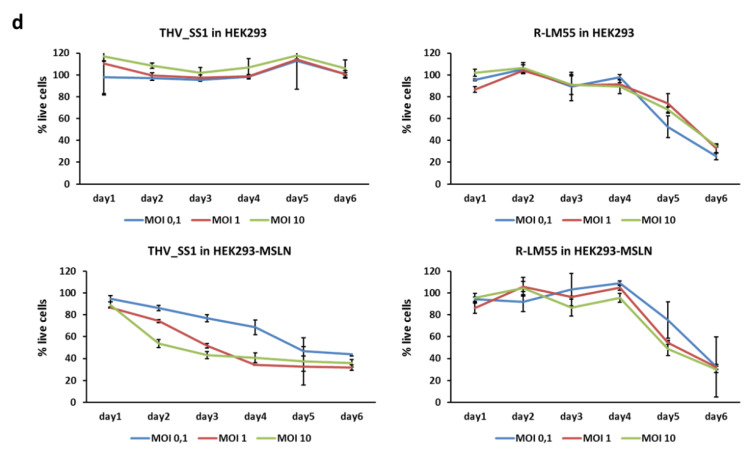
Cytopathic effect of THV_SS1 is tightly dependent on MSLN positive cells. (**a**) HEK293 cells (MSLN-) were infected from wild-type virus R-LM55, but not from THV_SS1 virus, confirming the stringency of the retargeting. (**b**) HEK293 cells were stably transduced with mesothelin protein and were efficiently infected (fluorescent positive cells) at different MOI_s_ (MOI from 0.001 to 1) by THV_SS1, up to 120 h post-infection. Bright-field images show the cytotoxic effect of THV_SS1 virus (round detached cells). (**c**) Mesothelin retargeting does not impact on viral replication in proficient cells, as THV_SS1 and parental R-LM55 viruses replicate equally well in HEK293-MSLN cell line. (**d**) The cytotoxic effect of R-LM55 and THV_SS1 in HEK293 and HEK293-MSLN was assessed by Alamar Blue. After 6 days from infection, R-LM55 reached 70% of cytopathic effect in both HEK293 cells (+/−MSLN), while THV_SS1 reached 70% of cytopathic infect only in HEK293-MSLN and maintains 100% of live cells in wild-type HEK293 cells.

Wild-type (Figure 3a) and MSLN^+^ HEK293 (Figure 3b) cells were infected with different dosage of THV_SS1 (MOI 0.001, 0.01, 0.1, 1 for HEK293-MSLN; MOI 1 for HEK293), and viral spread was monitored until reaching the infection of the whole cell monolayer. Wild-type R-LM55 HSV-1 was used as a positive control of infection (Figure 3a). The capacity of viruses in entry and spreading in the two cell lines was analyzed via fluorescence of eGFP-positive cells. As expected, HEK293 cells were efficiently infected by wild-type R-LM55, but resulted in being not infected by THV_SS1 virus, due to lack of MSLN as SS1 ligand (Figure 3a). In contrast, THV_SS1 resulted in a proficient infection of HEK293-MSLN cells, where a full cytopathic effect (CPE) was reached in a dose-dependent manner at the different time points post-infection (Figure 3b and Figure S1). In addition to virus entry, we investigated viral replication via detection of viral genome copies. The replication of THV_SS1 and of the parental R-LM55 was evaluated in HEK293-MSLN cell line; both wild-type and retargeted viruses replicated with similar effectiveness in the mesothelin-expressing cells (Figure 3c). The cytopathic effect was also evaluated in HEK293 and HEK293-MSLN cell lines infected with THV_SS1 and with the parental wild-type R-LM55 virus at different MOIs. The time-course analysis of cytotoxicity was carried out up to six days post-infection in the presence of 10% FBS. As expected, the wild-type virus R-LM55 infected and killed HEK293 as efficiently as HEK293-MSLN cell line, independently from human MSLN expression (Figure 3d). In contrast, the ability of THV_SS1 in infecting and killing cells resulted in being tightly dependent on human mesothelin display on cell surface, as the percentage of live HEK293 cells remained around 100% even at the highest dosage of THV_SS1 (10 MOI). The full virulence of the retargeted THV_SS1 virus was evident, as it lysed HEK293-MSLN cells in a similar fashion to wild-type R-LM55 virus assessed by Alamar Blue assay (Figure 3d) and bright field microscopy (Figure 3b).

**Figure 4:**
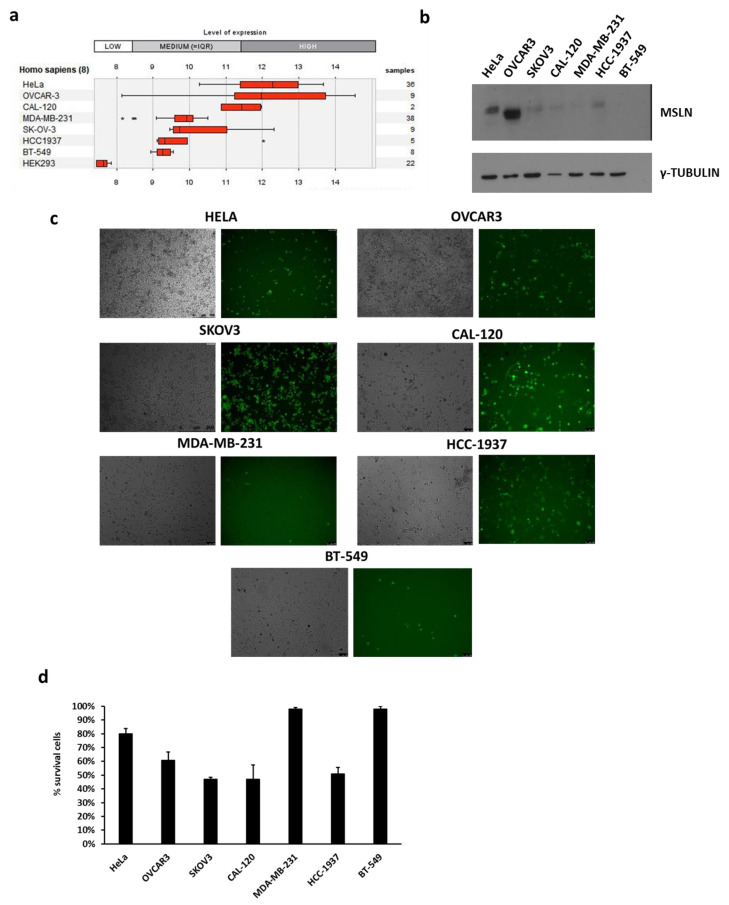
THV_SS1 infects breast and female reproductive system tumor cells in a MLSN-dependent manner. (**a**) Mesothelin expression assessed by GENEVESTIGATOR software by interrogating RNAseq repository of breast (CAL-120, HCC-1937, BT-549, MDA-MB-231) and female reproductive system (OVCAR3, HeLa, SKOV3) tumor cell lines. (**b**) MSLN expression in selected cell lines was evaluated by Western blot and gamma-tubulin was used as standard. (**c**) Infection and (**d**) cytotoxicity of THV_SS1 in tumor cell lines were assessed respectively by fluorescence/bright-field microscopy and trypan blue positive cells.

With the purpose to develop a targeted therapy for MSLN-positive breast and non-breast tumors, we tested our candidate oncolytic THV_SS1 in cancer cell lines of different origin. A panel of cell lines was selected, based on MSLN expression, as initially assessed by analysis of RNAseq repository database (GENEVESTINGATOR) [68]. HEK293 were reported as reference negative cell line. Cell lines derived from either female non-breast (SKOV3, OVCAR3 of ovarian cancer origin; HeLa for cervical cancer) or breast triple negative (CAL-120, BT-549, HCC1937, and MDA-MB-231) tumors were chosen as representative targets. HeLa, OVCAR3, and CAL-120 cell lines were, indeed, annotated with the highest MSLN expression. BT-549, MDA-MB-231 resulted slightly positive, while HEK293 cells were confirmed as negative for MSLN expression (Figure 4a). MSLN expression at the protein level by western blot analysis pretty well recapitulated the ranking of MSLN transcript expression in the selected panel of cell lines, with the exception of HeLa cells (Figure 4b). The data shown in Figure 4c highlight that all the selected tumor cell lines were infected by THV_SS1, although at different levels; considering that the cellular background may also affect viral infection, the obtained results were in good agreement with the relative MSLN expression data. A more detailed analysis, evaluating the relative percentages of living cells after infection, showed that residual viability ranged from 80 to 45% into the cell lines with remarkable MSLN expression. In particular, CAL-120, HCC-1937 TBNC cell lines and SKOV3 cell lines resulted the most infected (about 45% of residual cells), while OVCAR3 and HeLa were less (respectively 60 and 80%). FACS analysis of MSLN display on OVCAR3 cells showed a heterogeneous cell population (Supplementary Materials, Figure S2). Interestingly, the percentage of MSLN^+^ cells (30%) resulted remarkably similar to those killed by THV_SS1. The viability of BT-549 and MDA-MB-231 cell lines resulted almost unaffected by THV_SS1 infection.

### 2.3. Implementation of a Cell Platform for THV_SS1 Oncolytic Virus Production

**Figure 5:**
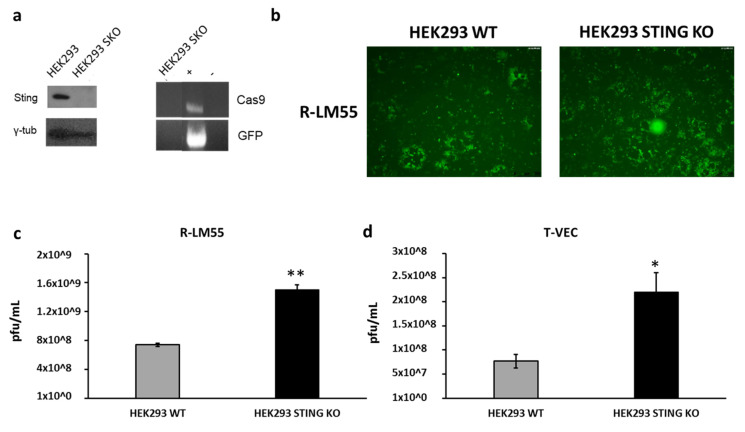
Generation of improved oncolytic virus producing cell line. (**a**) *STING* gene was knocked out in HEK293 cells as assessed by Western blot analysis; gamma-tubulin was used as standard. PCR screening confirmed the absence of eGFP and Cas9 residues in genomic DNA; Cas9/eGFP-encoding vector was used as positive control (C+) and genomic DNA from parental HEK293 cell was used as negative control (C−). (**b**) R-LM55 viral spread enhancement was demonstrated in HEK293 STING KO cell line compared to HEK293 WT cell line. (**c**,**d**) Enhancement viral yield was demonstrated on HEK293 STING KO vs. parental infected with R-LM55 and T-VEC viruses. The statistical significance was calculated by Student’s t-test comparing HEK293 STING KO and HEK 293 WT and resulted *p* < 0.005 ** and *p* < 0.05 *, respectively, for R-LM55 and T-VEC productivity.

The nucleic acid sensing pathway based on STING protein is involved in recognition of different species of both DNA and RNA viruses [69]. Accordingly, many viral species have developed molecular mechanisms to inhibit STING axis at different levels of the pathway cascade, to evade interferons-mediated viral clearance [70,71,72,73,74,75,76,77,78]. The conservation of these escape mechanisms across different viruses underlines the key role that *STING* gene plays in host and microbe interactions [79,80]. We have recently described how the DNA sensing regulator STING is crucial to restrict the replication of an oncolytic HER2-retargeted *Herpes* virus [47]. Based on this evidence, we speculated that the functional inactivation of *STING* in a suitable producing cell line could improve the yield of oncolytic vectors, including retargeted THV_SS1. In order to eliminate any possible bias by the endogenously produced MSLN, we decided to use HEK293 cells, physiologically negative for the protein. In fact, we demonstrated interference of high levels of SMRP to THV_SS1 infection, thus, a supraphysiological shedding of SMRP in long-term cell cultures used for virus production could arise, decreasing the actual viral yields. Guide RNAs (gRNAs) were designed, to target the first coding exons of *STING* by CRISPR/Cas9 system. Among several *STING* knock-out subclones, we selected the best one, based on the absence of Cas9 cloning residues (Figure 5a). The usefulness of *STING* knock-out was assessed in a proof-of-concept experiment using the wild-type R-LM55 *Herpes* virus and the clinically validated T-VEC virus. The inactivation of *STING* resulted in an enhanced viral spread (Figure 5b) and in a significant improvement in viral yields (Figure 5c,d).

**Figure 6:**
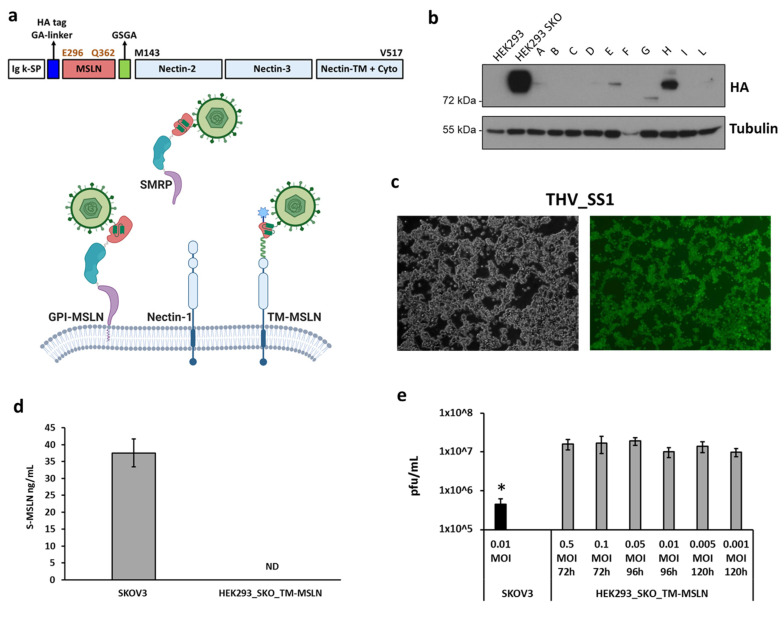
Generation of oncolytic virus HEK293 STING KO producing cell line stably expressing an engineered non-secretable MSLN. (**a**) In the upper part is shown the chimeric MSLN-Nectin construct that consists of: Ig-κ signal peptide (white box), HA tag plus GA-based linker (blue box), the N-terminus 66 amino acid fragment of MSLN (E296-Q362) (red box), GSGA-based linker (green box), the C-terminus of Nectin-1 including the transmembrane and intracellular tail (M143-V517) (light-blue boxes). In the bottom part is shown a cartoon model that depicts the interaction of THV_SS1 with GPI-MSLN, SMRP, TM-MSLN proteins. Created by Biorender. (**b**) Western blot screening for HA tag to find TM-MSLN protein positive clones; clone H was chosen, and gamma-tubulin was used as standard. (**c**) HEK293_SKO_TM-MSLN cell line were efficiently infected by THV_SS1 at 0.005 MOI. (**d**) The secreted MSLN in SKOV3 and HEK293_SKO_TM-MSLN was quantified by ELISA assay. (**e**) The productivity of THV_SS1 was evaluated in SKOV3 at 0.01 MOI and in HEK293_SKO_TM-MSLN at different MOI (from 0.001 to 0.5). The statistical significance was calculated by Student’s *t*-test and resulted as not significant, comparing the different MOI in HEK293_SKO_TM-MSLN cells (0.5, 0.1, 0.05, 0.01, 0.005, 0.001 MOI). The difference between SKOV3 and HEK293_SKO_TM-MSLN resulted as significant (*p* < 0.05 *).

We next decided to exploit HEK293 *STING* KO cells as a platform for THV-SS1 production. As HEK293 *STING* KO cells do not express MSLN, its transduction was preparatory to render these cells proficient for THV-SS1 infection. Thus, to overcome the potential drawback of supraphysiological shedding of SMRP, which can be occurring during virus production, we designed a non-secretable, non-oncogenic human mesothelin variant (transmembrane-MSLN, TM-MSLN) by fusing the minimal domain of MSLN recognized by SS1 scFv to the C-terminus of transmembrane human Nectin-1 (Figure 6a). With this aim, the N-terminal 66 amino acid-long fragment of MSLN (E296-Q362) [81] was inserted downstream an Ig signal peptide (SP), and upstream the C-terminus of Nectin-1, including the transmembrane and intracellular tail (M143-V517). Two linkers spaced MSLN fragment from SP and Nectin-1 to improve the accessibility of THV-SS1. The expression of chimeric TM-MSLN was evaluated by Western blot analysis recognizing the HA tag enclosed at the N-terminus of TM-MSLN (Figure 6a). Among several clones, we selected the most expressing one (clone H, hereinafter referred as HEK293_SKO_TM-MSLN) for further characterizations (Figure 6b). The possible interference by secreted, soluble mesothelin, on THV_SS1 spread and productivity, was finally evaluated in the HEK293_SKO_TM-MSLN cells. Thus, a very low multiplicity of infection MOI (0.005) was used, to infect HEK293_SKO_TM-MSLN cells; as a result (Figure 6c), the whole cell monolayer resulted infected at 120 h post infection, demonstrating the absence of competition by SMRP potentially accumulated in the cell medium. Consonant to this finding, SMRP was not detectable in conditioned media from HEK293_SKO_TM-MSLN cells, even at 4 days from seeding (Figure 6d). Finally, viral yield was evaluated for THV_SS1 in HEK293_SKO_TM-MSLN at different viral dosages (MOI range from 0.001 to 0.5). Cell lysates were harvested when full cytopathic effect was reached (72-, 96-, 120 h respectively for 0.5 and 0.1, 0.05 and 0.01, 0.005 and 0.001 MOI) and infectious viral particles were quantified by plaque assay (Figure 6e). The THV_SS1 production in HEK293_SKO_TM-MSLN was compared to the THV_SS1 production in the cell line expressing wild-type mesothelin (SKOV3), resulting in a 40-fold improvement in viral particles (Figure 6e). Thus, the combination of *STING*-knockout and high expression levels of non-secretable MSLN by the modified HEK293 cells represented an appropriate implementation for improved productivity of the THV_SS1 virus.

## 3. Discussion

During the last 40 years, the deep knowledge of the human genome, accompanied by the development of novel technologies for genome editing of viruses, has revolutionized the modern medicine. The use of viral vectors for gene therapy, vaccine delivery, and oncolytic viruses is now a reality, with thousands of clinical trials completed, ongoing, or approved worldwide [1,3,82,83]. Oncovirotherapy with retargeted viruses is revealing a promising potential for the treatment of many cancer indicators, including breast cancer [7,23,47,66,84]. We recently characterized preclinical models of HER2-positive tumors in the framework of combination with checkpoint inhibitors [7,23,47]. Among breast cancer, triple-negative tumors are characterized by lack of canonical breast receptors (i.e., HER2, ER, PR) amenable by targeted therapeutics. So, a potential to complement innovative therapeutic tools for these neoplasms, by additional retargeting approaches of herpetic viruses, is a current requirement. Accordingly, in this study we developed novel HSV-1 based oncolytic viruses targeting mesothelin, a TAA frequently expressed in triple-negative breast cancer, as well as in additional neoplasms, including the orphan disease mesothelioma. Our proof-of-concept study took advantage from available antibody sequences with a strong binding affinity to two different regions of mesothelin (region I, region III). Interestingly, we proved that the docking of retargeted HSV-1 to membrane proximal domain of target TAA does not necessarily improve infection but, on the contrary, could hamper virus-host interaction presumably due to steric hindrance. The Amatuximab derived SS and SS1 scFv_s_ targeting region I of human MSLN were highly effective in mediating THV infection of MSLN^+^ human cancer cells. Importantly, the infection was unaffected by the presence of soluble mesothelin, SMRP, at a concentration range similar to that exhibited by patients affected by mesothelin-expressing tumors. The infective potential of the MSLN-THVs resulted in being strictly dependent on MSLN expression; analysis of a panel of triple-negative breast cancer cells, and of cells from cervical and ovarian tumors, showed good agreement of residual cell viability after infection with the expression levels of mesothelin. Namely, triple-negative cell lines with higher levels of mesothelin were effectively killed by MSLN-THV, while the cell lines showing the lowest levels of MSLN expression were refractory to the retargeted virus; this may also take into account the natural variability of different cellular backgrounds, in terms of susceptibility to viral infection. The most effective mesothelin targeted herpes virus (THV_SS1) described in this study was further implemented by encoding murine IL12 immunostimulatory cytokine as the latter was recently demonstrated to synergize with HER2-retargeted herpes virus in mediating systemic anti-tumor immunity [23,66]. The availability of an unarmed, and of the corresponding IL12 armed viruses, will allow to initiate preclinical studies, evaluating the suitability of these oncolytic constructs to co-operate with immune checkpoint blockade in human mesothelin-tolerant, immunocompetent murine models.

Moreover, with the aim to implement a cell platform to improve viral yield for manufacturing, we exploited the *STING*-based antiviral pathways. The choice for *STING* inactivation was related to the intrinsic features for this gene, which is highly efficient in restricting viral replication, so that most of the known viral species have evolved sophisticated *STING*-specific escape mechanisms [54]. As antiviral pathways are often inactivated in cancer cells, to restrict the oncolytic potential to tumor tissues, most of the OVs harbor attenuating mutations that affect their anti-viral escape mechanisms (e.g., HSV-1 deletion in *γ34.5* gene). Based on this evidence, we functionally inactivated *STING* into a producing cell line, taking the brakes off for viral replication. We validated the concept of *STING* knock-out for viral manufacturing of both wild-type, unattenuated HSV-1, as well as for an oncolytic virus with molecular features almost identical to those of the clinically approved T-VEC, talimogene laherparepvec, Imlygic (*34.5*-/*47*-/GM-CSF). The higher enhancement in viral yield of T-VEC, compared to wild-type (R-LM55) HSV-1 was presumably due to the lack of STING-antagonist *34.5* gene in T-VEC genome, which renders wild-type viruses more susceptible to STING restriction [78]. Thanks to the broad effect of STING on many viral species, we presumed that the proof-of-concept of *STING* knock-out for viral manufacturing could be applied immediately, not only to oncolytic viruses, but also into the field of viral vectors for gene therapy and genetic vaccines. This could be of great interest for manufacturing of recombinant viral vector vaccines targeting pandemic viruses including SARS-CoV-2 currently responsible for COVID-19 pandemic [85,86,87].

The *STING* knock-out cell line was further adapted to production of the SS1-THV, by designing a novel non-secretable MSLN to face the potential limitation linked to the accumulation of soluble MSLN (SMRP) in the supernatant of cells for MSLN-THV manufacturing. In fact, while the presence of SMRP in the tumor microenvironment is not expected to interfere with the oncolytic potential of MSLN-based retargeted viruses, it could render suboptimal their productivity and scalability for clinical applications. To that end, we designed and stably expressed a chimeric MSLN receptor by fusing the scFv-targeted minimal domain of MSLN to a transmembrane cellular adhesion molecule.

In conclusion, our study describes the suitability of our proof-of-principle, proposing MSLN as a good candidate for retargeting by oncolytic viruses. The availability of these novel MSLN-targeted herpetic THVs, combined to the generation of the cell platform we developed for viral manufacturing, will allow a rapid translation to preclinical triple-negative breast cancer models.

## 4. Materials and Methods

### 4.1. Cell Culture, Manipulation, and Characterization

SKOV3, BT-549 cell lines were cultured in RPMI 1640 Medium GlutaMAX^TM^ Supplement; CAL-120, MDA-MB-231, HEK293, HEK293 STING KO, HEK293-MSLN, and HEK293_SKO_TM-MSLN cell lines were cultured in Dulbecco’s Modified Eagle Medium (DMEM); HCC-1937 cell line was cultured in Iscove’s Modified Dulbecco’s Medium (IMDM). All media were supplemented with 10% heat-inactivated fetal bovine serum (FBS) (except for HCC-1937 cell line with 20% FBS), 50 UI/mL penicillin, 50 μg/mL streptomycin, 2 mM glutamine (except for HCC-1937, CAL-120 and MDA-MB-231 cell lines with 4 mM glutamine). HEK293-MSLN (bulk population) and HEK293_SKO_TM-MSLN media were supplemented with hygromycin for human MSLN transgene expression. BT-549 cell line was supplemented with 10 mM HEPES. All the reagents for cell culturing were from Gibco^TM^, Thermo Fisher Scientific (Waltham, MA, USA). Cell lines were purchased from the American Type Culture Collection (ATCC) and from CEINGE Biotecnologie Avanzate cell bank facility.

*Sting* knockout was carried out by CRISPR/Cas9 with gRNA reported in Table 1. The presence of eGFP and Cas9 was evaluated by PCR with oligonucleotide sequences reported in Table 1. Knockout was assessed by Western blot analysis as reported in our previous publication [88]. Briefly, the cell pellets were lysed in a buffer containing 20 mM Tris-HCl (pH 7,5), 100 mM NaCl, 0.5% Triton X-100, 50 mM NaF, and 10 mM glycerophosphate in the presence of protease inhibitors (Complete™ Protease Inhibitor Cocktail, Roche, Basel, Switzerland). The extracts were clarified by centrifugation at 12,000 rpm at 4° after 20 min in ice. Protein concentration was determined by Bradford colorimetric assay (Bio-Rad Protein Assay Dye Reagent Concentrate, Biorad, Hercules, CA, USA). Western blot analysis was carried out on cellular lysates; the latter were resolved on 4–12% SDS-PAGE gels (Invitrogen, Carlsbad, CA, USA) and transferred to membrane (Invitrogen, Carlsbad, CA, USA). Filters were probed with anti-MSLN, anti-STING, and anti-HA antibodies.

FACS analysis was performed according to the procedure previously described [89]. Briefly, 1 *×* 10^6^ OVCAR3 cells were detached by using PBS EDTA 5 mM and washed 2 times with PBS. Cells were stained with anti-MSLN antibody and were analyzed and monitored by cell-sorter Becton Dickinson FACSAria.

To design a non-secretable human mesothelin that retained the interaction with scFv of interest, a fusion protein was engineered. As the major cleavage sites of MSLN to generate SMRP are near the cell membrane [90], the chimeric construct was engineered by fusing nectin-1 C-terminus to N-terminus of human mesothelin interacting with SS and SS1 scFv_s_.

### 4.2. Modifications of BAC-HSV-1 Vectors

The sacB/ampR/lacZ recombineering system was exploited to modify HSV-1 vectors as previously reported [7]. THV_SD1, THV_SS, and THV_SS1 were generated starting from the wild type strain F HSV-1 (GenBank accession number: GU734771.1) derivative R-LM55 containing BAC insertion in UL3-UL4 intergenic region. The second generation THV was generated starting from THV_SS1 virus, with mIL-12 insertion in US1-US2 intergenic region [66].

Briefly, a DNA fragment containing the sacB/ampR/lacZ selection cassette with 40 base-pair homology arms to the region to be engineered was inserted through electroporation into SW102 containing the BAC-HSV-1 (R-LM55 or THV_SS1). SW102 cells were plated on LB agar plus 12.5 μg/mL chloramphenicol, 20 μg/mL ampicillin, 80 μg/mL X-gal, and 200 μM IPTG; the blue colonies were cultured in LB medium and DNA was extracted.

The second step of recombineering was performed by electroporation of the first-step derivate SW102 cells with a homology arms flanked DNA fragments containing the antibody fragments (SD1, SS, and SS1) or mIL-12. The negative selection was performed on plates containing sucrose.

A detailed list of oligonucleotides is reported in Table 1.

### 4.3. Viral Rescue, Production, Titration, and Real Time PCR Analysis

The viruses were produced and titrated in SKOV3 cells according to the procedure previously described [7]. Briefly, for viral rescue SKOV3 cells were transfected with BAC-HSVs DNA by Lipofectamine 2000 (Life Technologies, Carlsbad, CA, USA) and grown up until full cytopathic effect (CPE) was reached.

Viral titration was made by plaque assays by 10-fold scaling dilutions and staining with GIEMSA 150 h post infection.

Viral genome copies replication was titrated by TaqMan RealTime PCR (Taqman universal PCR mastermix, Applied Biosystems, Foster City, CA, USA) from cell lysates. Briefly, viral samples were treated with RNase-free, DNase I recombinant enzyme (Roche, Basel, Switzerland) to eliminate envelope-free viral DNA. Viral DNA was thus extracted from enveloped HSV-1 particles by SDS 0.1% (*w*/*v*, final concentration) and proteinase K (Roche, Basel, Switzerland). The extracted viral particles were diluted and analyzed by TaqMan RealTime PCR according to the manufacturer’s recommendations (oligoes and probe in Table 1).

### 4.4. Tropism of the Recombinant Viruses

Amatuximab (α-MSLN) was used to verify the neutralization of THV_SS and THV_SS1 infection. Amatuximab was produced by ourselves by subcloning the variable region of SS1_scFv into Fc-expressing vectors as described previously [91,92]. Antibody was produced by transfection of the expression vectors into the production enhanced cell line HEK293_ES1 [93] expressing the long non-coding SINEUP [94] targeting heavy and light chains signal peptide on mRNAs. Monoclonal antibody was purified from cell supernatant by protein A affinity chromatography. For neutralization assay, increasing concentration of purified Amatuximab (from 150 to 0 μg/mL) were used to pre-incubate SKOV3 cell line at 37°. After 2 h, cells were infected with THV_SS and THV_SS1 at MOI 1 (pfu/cell) adding the virus mix into the conditioned medium. Pictures and number of eGFP positive cells were analyzed after 24 h post infection.

### 4.5. Quantification of Soluble Proteins

The interference of soluble mesothelin (SMRP) during the THV_SS and TV_SS1 infection was investigated through the dosage of the extracellular release of SMRP in the medium. SKOV3 or HEK293_SKO_TM-MSLN cells were seeded in 12-well plates and supernatant were collected from 24 to 96 h post seed after centrifugation at 200× *g* for 5 min to remove debris. SMRP was measured by LEGEND MAX™ Human Mesothelin ELISA Kit (Biolegend, San Diego, CA, USA) according to the manufacturer’s protocol. The same supernatants containing SMRP were used to precondition and infect fresh SKOV3 cells with THV_SS and THV_SS1 at MOI 0.1 pfu/cell. The size of viral spread plaques was analyzed 48 h post infection by fluorescence microscope (DMI4000B, Leica, Wetzlar, Germany).

The validation of the transgene production by second generation THV was investigated through the extracellular release of the cargo (mIL-12). SKOV3 cells were seeded in 12-well plates and infected with THV_SS1-IL12 at MOI 0.1 pfu/cell. The supernatants were collected 48, 72, and 96 h post infection and debris were removed by 5′ centrifugation at 200× *g*. Secreted mIL-12 was measured by ELISA Kit (Invitrogen, Carlsbad, CA, USA) according to the manufacturer’s protocol.

### 4.6. Cytotoxicity Assay

On day −1, HEK293 and HEK293-MSLN cells were seeded in 96-well plates in the presence on 10% FBS. To avoid cellular stress response related to 6 days culturing without medium refresh, 1e5 cells *per* well were seeded to obtain a confluence of 60% on day 0. Cells were infected with R-LM55 and THV_SS1 at MOI 0.1, 1 and 10 pfu/cell; after 2 h incubation, conditioned media were replaced with 150 µL of fresh DMEM supplemented with 10%FBS and 2 mM GlutaMAX Gibco^TM^, Thermo Fisher Scientific (Waltham, MA, USA). AlamarBlue^®^ (Biorad, Hercules, CA, USA) was added to the culture (10 μL/well) and incubated 4 h at 37 °C from day 1 to day 6 after infection. The plates were read at 570 nm and 600 nm with EnVision Multimode Plate Reader (PerkinElmer, Waltham, MA, USA). The relative cytotoxicity was expressed as the percentage difference of infected over not infected cells.

### 4.7. Figures and Statistical Analysis

Images were created using Biorender software, a web-based tool useful for generating biomedical research drawings [95].

GraphPad Prism was used to perform the Student’s *t*-test statistical analysis. The significance was reported according to the following code *p* < 0.05 *; *p* < 0.005 **.

## 5. Patents

The concept of Sting KO for enhancement viral production has been filed in a patent.

## Figures and Tables

**Table 1:** Oligonucleotide sequences.

Name	Sequence
CAS9_Fwd	5′-gctctttgatgccctcttcg-3′
CAS9_Rev	5′-gctgaccctgacactgtttg-3′
GFP_Fwd	5′-cacgacttcttcaagtccgc-3′
GFP_Rev	5′-ggtgttctgctggtagtggt-3′
hSting gRNA1_Fwd	5′-caccggtgacccctgggacacggga-3′
hSting gRNA1_Rev	5′-aaactcccgtgtcccaggggtcacc-3′
hSting gRNA2_Fwd	5′-caccggctgggactgctgttaaac-3′
hSting gRNA2_Rev	5′-aaacgtttaacagcagtcccagcc-3′
Step I gD_Fwd	5′-agtgggcctccatggggtccgcggcaaatatgccttggcgacccctatttgtttatttttct-3′
Step I gD_Rev	5′-tggggggctggaacgggtccggtaggcccgcctggatgtgttatttgttaactgttaattgtc-3′
Step II gD_SD1_Fwd	5′-catggggtccgcggcaaatatgccttggcgcaggtgcagctggtgcagtc-3′
Step II gD_SS/SS1_Fwd	5′-agtgggcctccatggggtccgcggcaaatatgccttggcgcaggttcagctgcagcagtc-3′
Step II gD_SD1/SS/SS1_Rev	5′-tggggggctggaacgggtccggtaggcccgcctggatgtgagatcctccgcttccgctgc-3′
Step I US1/US2_Fwd	5′-cgtttgtcccagcgtcttaatggcgggaagacccctatttgtttattttt-3′
Step I US1/US2_Rev	5′-ccatgtacgcgtggtctgtttctctccgccttatttgttaactgttaatt-3′
Step II US1/US2_mIL12_Fwd	5′-cgtttgtcccagcgtcttaatggcgggaagacattgattattgactagtt-3′
Step II US1/US2_mIL12_Rev	5′-ccatgtacgcgtggtctgtttctctccgccgccatagagcccaccgcatc-3′
Taqman DNApol_Fwd	5′-catcaccgacccggagagggac-3′
Taqman DNApol_Rev	5′-gggccaggcgcttgttggtgta-3′
Taqman Probe	FAM-ccgccgaactgagcagacacccgcgc-Tamra

## Data Availability

Data sharing not applicable.

## Supplementary Materials

The following are available online at https://www.mdpi.com/1422-0067/22/2/477/s1. Supplementary Figure S1. Replication and cytopathic effect of THV_SS1 in MSLN positive cells. HEK293MSLN cells were efficiently infected (fluorescent positive cells) by THV_SS1 and reach a complete full cytopathic effect (round detached cells) in dose- and time-dependent manner. Supplementary Figure S2. FACS analysis of MSLN display on OVCAR3 cells surface. OVCAR3 cells were stained with II Ab or with anti-human MSLN plus II Ab. The 30% of cell population resulted as highly positive for MSLN.

## Abbreviations

MSLN	Mesothelin
THV	Targeted Herpes Virus
STING	Stimulator of Interferon Genes
OV	Oncolytic virus
scFv	Single Chain Fragment variable
SMRP	Soluble Mesothelin Related Protein
