# Supplementary material for: Generation of a Novel Mesothelin-Targeted Oncolytic Herpes Virus and Implemented Strategies for Manufacturing
Source: Int J Mol Sci. 2021 Jan 6;22(2):477. doi: 10.3390/ijms22020477 (PMC7825047; doi:10.3390/ijms22020477)
Supplement: Supplementary file 1 [file ijms-22-00477-s001.pdf]

Supplementary Figures

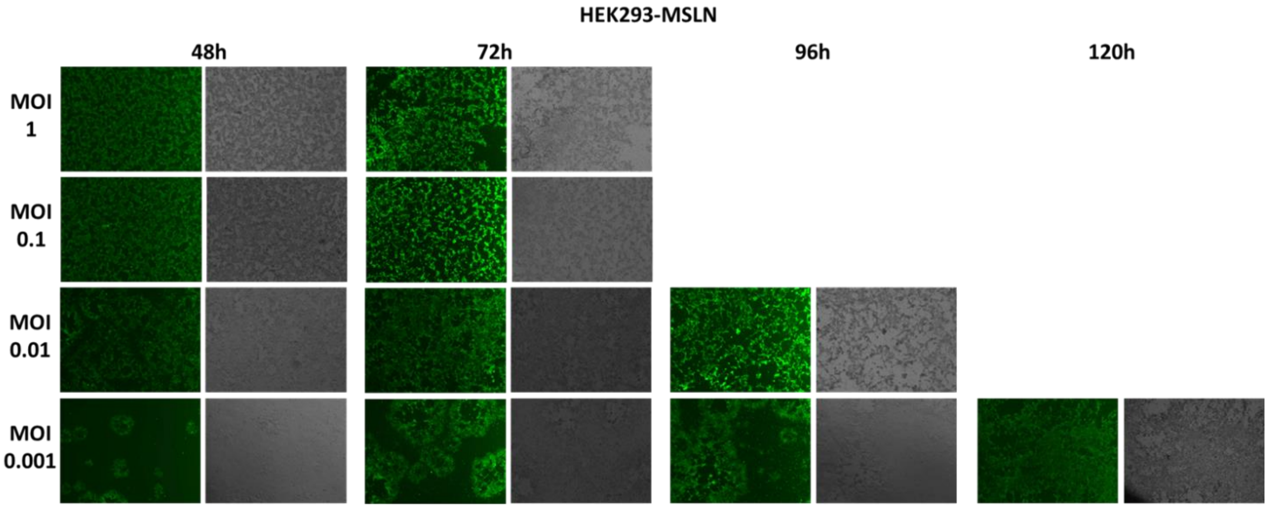

Figure S1

Supplementary Figure S1. Replication and cytopathic effect of THV_SS1 in MSLN positive cells. HEK293MSLN cells were efficiently infected (fluorescent positive cells) by THV_SS1 and reach a complete full cytopathic effect (round detached cells) in dose- and time-dependent manner.

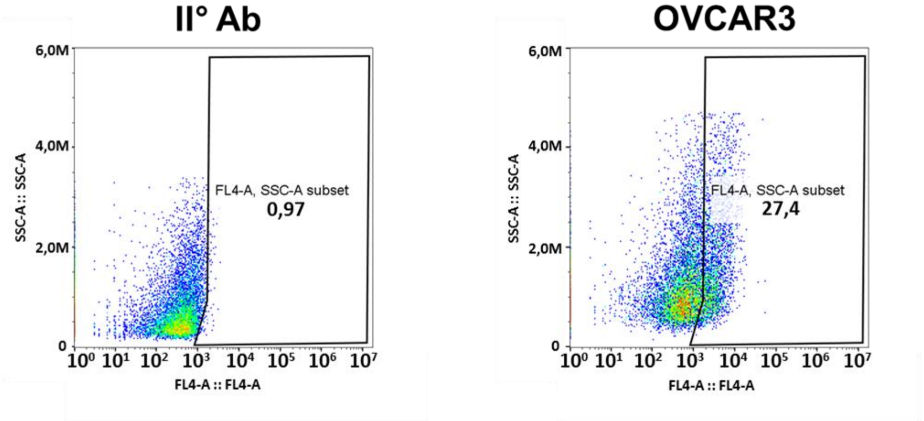

Figure S2

Supplementary Figure S2. FACS analysis of MSLN display on OVCAR3 cells surface. OVCAR3 cells were stained with II Ab or with anti-human MSLN plus II Ab. The 30% of cell population resulted as highly positive for MSLN.
